# Radiotherapy combined with TLR7/8 activation induces strong immune responses against gastrointestinal tumors

**DOI:** 10.18632/oncotarget.3081

**Published:** 2014-12-31

**Authors:** Sebastian Schölch, Conrad Rauber, Alexandra Tietz, Nuh N. Rahbari, Ulrich Bork, Thomas Schmidt, Christoph Kahlert, Uwe Haberkorn, Mark A. Tomai, Kenneth E. Lipson, Rafael Carretero, Jürgen Weitz, Moritz Koch, Peter E. Huber

**Affiliations:** ^1^ Department of Gastrointestinal, Thoracic and Vascular Surgery, Medizinische Fakultät Carl Gustav Carus, Technische Universität Dresden, 01307 Dresden, Germany; ^2^ Department of General, Gastrointestinal and Transplant Surgery, University Hospital Heidelberg, Ruprecht-Karls-Universität Heidelberg, 69120 Heidelberg, Germany; ^3^ CCU Molecular and Radiation Oncology, German Cancer Research Center, 69120 Heidelberg, Germany; ^4^ Department of Radiation Oncology, University Hospital Center, 69120 Heidelberg, Germany; ^5^ Division of Nuclear Medicine, Department of Radiology, University Hospital Heidelberg, Ruprecht-Karls-Universität Heidelberg, 69120 Heidelberg, Germany; ^6^ 3M, Inc., St. Paul, Minnesota 55121, USA; ^7^ Fibrogen Inc., San Francisco, California 94158, USA; ^8^ Division of Molecular Immunology, German Cancer Research Center, 69120 Heidelberg, Germany

**Keywords:** colorectal cancer, pancreatic cancer, TLR7/8 ligand, radiotherapy, immunotherapy

## Abstract

In addition to local cytotoxic activity, radiotherapy may also elicit local and systemic antitumor immunity, which may be augmented by immunotherapeutic agents including Toll-like receptor (TLR) 7/8 agonists. Here, we investigated the ability of 3M-011 (854A), a TLR7/8 agonist, to boost the antigen-presenting activity of dendritic cells (DC) as an adjuvant to radiotherapy. The combined treatment induced marked local and systemic responses in subcutaneous and orthotopic mouse models of colorectal and pancreatic cancer. *In vitro* cytotoxicity assays as well as *in vivo* depletion experiments with monoclonal antibodies identified NK and CD8 T cells as the cell populations mediating the cytotoxic effects of the treatment, while *in vivo* depletion of CD11c^+^ dendritic cells (DC) in CD11c-DTR transgenic mice revealed DC as the pivotal immune hub in this setting. The specificity of the immune reaction was confirmed by ELISPOT assays. TLR7/8 agonists therefore seem to be potent adjuvants to radiotherapy, inducing strong local and profound systemic immune responses to tumor antigens released by conventional therapy.

## INTRODUCTION

While considerable progress has been made in the therapy of colorectal cancer (CRC), the prognosis of metastatic CRC is still often unfavorable [1]. The prognosis for pancreatic cancer (pancreatic ductal adenocarcinoma, PDAC) is even worse [2]. Traditional systemic therapies are often unable to eradicate all tumor cells and hence usually cannot provide a cure. In contrast, immunotherapy may be able to definitively eradicate the tumor as it does not target the tumor directly but induces an immune response to tumor antigens in general.

Among the currently most successfully used immunotherapeutics are imidazoquinoline derivatives, most notably imiquimod (Aldara^®^ cream), a selective TLR7 agonist, which can be topically used in malignant and premalignant skin lesions and shows considerable response rates [3]. TLR are pattern recognition receptors involved in the sensing of structurally conserved molecules derived from microbes and therefore predominantly expressed on immune cells, most notably APC (such as DC and macrophages) and effector cells of both the innate and adaptive immune system (such as macrophages, NK cells, B cells and cytotoxic T cells). Upon antigen contact and TLR activation, these cells show increased proliferation, cytokine secretion, costimulatory molecule expression, phagocytic activity and antigen presentation [4]. The agent used in the present work, 3M-011, an agonist for TLR7/8, is soluble in aqueous buffers and can therefore be administered systemically [5,6]. 3M-011 does not activate mouse TLR8, thus acting as a TLR7 agonist only in the murine setting [5].

Tumor vaccines generally aim to induce antigen-specific humoral (B cell-based) or cytotoxic (T cell-based) immune responses. As the response to the antigen is usually insufficient to induce clinically relevant and durable immune responses, numerous adjuvants have been evaluated [7]. TLR agonists strongly increase APC activity and priming of the adaptive immune system, resulting in augmented responses to the vaccine and thus seem to be suitable adjuvants [8]. TLR7 and TLR8 are expressed in plasmacytoid (pDC) and myeloid (mDC) dendritic cells, respectively. Upon activation, mDC produce various proinflammatory cytokines, show increased antigen phagocytosis and processing and prime CD8 and CD4 T cells [9]. pDC produce high amounts of type 1 INF and thus boost the activity of CD8 T cells and NK cells [10].

Radiotherapy is a mainstay in the treatment of many solid tumors including gastrointestinal tumors. Data from multiple cancer models have provided sufficient evidence to propose a paradigm shift, whereby some of the effects of ionizing radiation are recognized as contributing not only to local tumor control but also to local and systemic antitumor immunity [11-14]. The underlying radiation effects include the induction of a pro-inflammatory microenvironment within the tumor, as well as the release of tumor antigens which can subsequently be processed and presented by APC, thus acting as an intrinsic tumor vaccination. As opposed to classical tumor vaccination, which usually induces an immune reaction against a single, tumor-specific antigen, the radiation-induced immune reaction is directed against multiple epitopes. As tumors are immensely heterogeneous, this polyclonal immune response may prevent tumors from evading eradication by downregulating the antigens targeted by the immune system. Both ionizing radiation and pro-inflammatory drugs such as TLR ligands may therefore be able to synergistically contribute to the eradication of tumors by the immune system [15,16].

**Fig.1:**
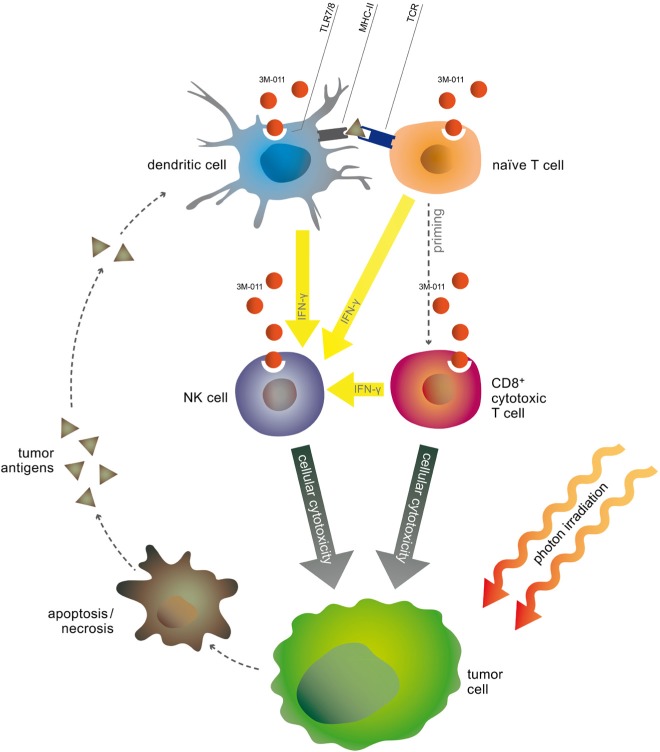
Scheme of putative synergistic effects of the therapy combination

In the present study, we therefore aimed to investigate the effects of a combination of the TLR7 agonist 3M-011 and fractionated radiation therapy. We hypothesized that the TLR ligand might increase processing and presentation of antigens released by concomitant radiation, therefore acting as an adjuvant to the vaccination effect of radiotherapy (Fig. 1). We demonstrated the effectiveness of the therapy combination and identified the involved immune cell populations and subsequently investigated their role by selective *in vivo* depletion.

## RESULTS

As previous reports had indicated an involvement of TLR signaling in tumorigenesis [17], we first evaluated the effects of the TLR7/8 agonist on various tumor cell lines. We therefore performed WST proliferation assays on murine and human tumor cell lines of CRC and PDAC and found that 3M-011 had only minimal and divergent effects on tumor cell proliferation *in vitro* and only at concentrations unattainable *in vivo* (Supplemental Fig. 1). Of note, there was no direct radiosensitizing effect of 3M-011.

### When combined with ionizing radiation, 3M-011 effectively inhibits the growth of syngeneic colorectal and pancreatic tumors and can cause complete remissions

**Fig.2:**
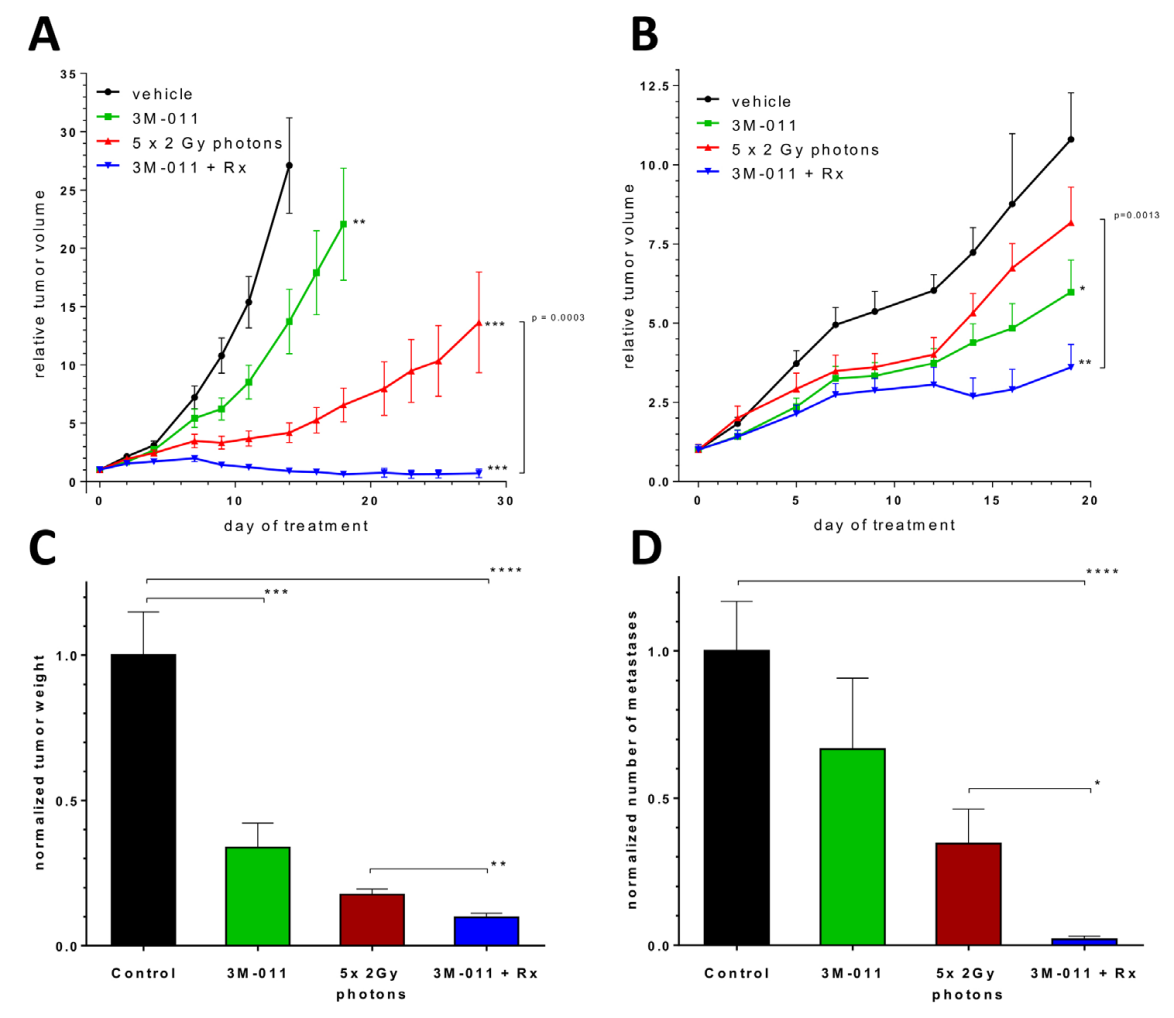

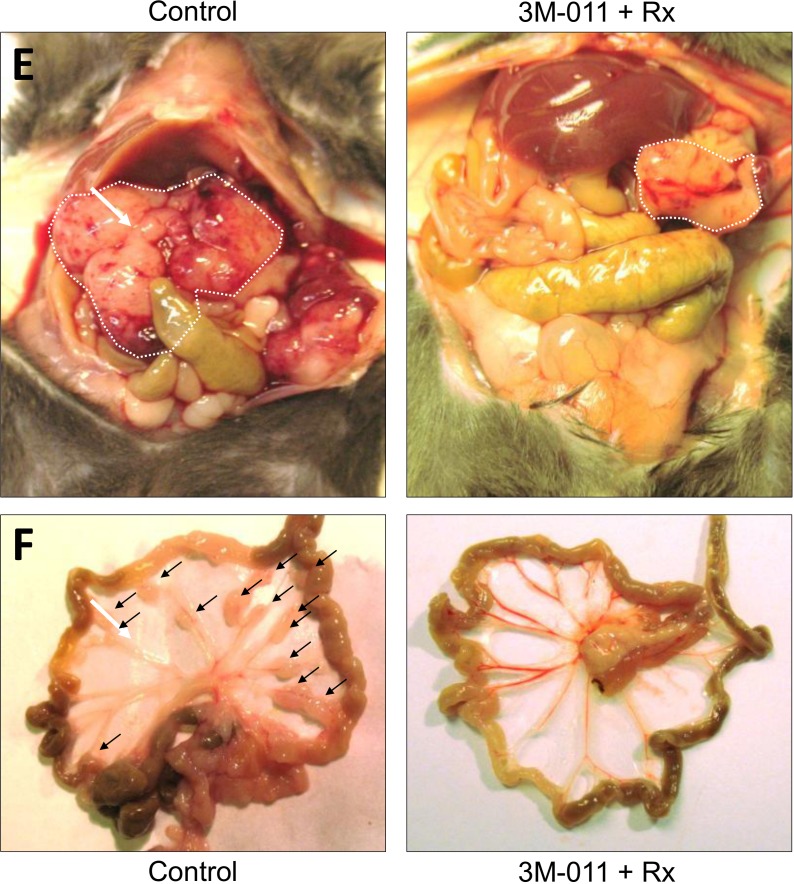
*In vivo* therapeutic experiments A. Growth curves of subcutaneous CT26 CRC homografts. B. Growth curves of subcutaneous Panc-02 PDAC homografts. C-D. Tumor weight and number of mesenteric lymph nodes at day 11 after orthotopic inoculation of Panc-02 PDAC tumor cells. E-F. Exemplary necropsy images (top: abdominal cavity, tumor indicated by white dotted line, bottom: mesentery, metastases indicated by black arrows) of mice with orthotopic PDAC tumors at day 11 after inoculation. *Black = Control; green = 3M-011; red = Rx (5×2 Gy photons); blue: 3M-011 + Rx.* All values represent mean ± SEM. For clarity reasons, comparisons to the control group are indicated by asterisks without brackets.

To investigate the effects of the TLR7/8 agonist in combination with classical fractionated photon radiotherapy *in vivo*, we induced tumor homografts by subcutaneous implantation of CT26 tumor fragments in immunocompetent BALB/c mice. Treatment with 3M-011, fractionated local irradiation (Rx, targeted to the subcutaneous tumor) or the combination of both started when the tumors were established, growing and about 50 mm³ in size. Vigorous tumor growth required the euthanasia of vehicle-treated mice in the control group by day 14 after the start of treatment. Significantly reduced tumor volume was noted in the 3M-011 monotherapy group (p<0.01, Fig. 2A). Unsurprisingly, the 5× 2 Gy radiotherapy regime also reduced tumor volumes (p<0.001) and induced complete remissions in ~20% of the animals. The combination of both 3M-011 and radiation showed complete remission of the tumors in 50% of the mice (p<0.001 vs. control group, p=0.0003 for Rx + 3M-011 vs. Rx alone). The combination group also showed at least additive activity in local control rates, yielding local control in 50% of the mice (х² = 11.2, p<0.01 for two-sided tests). In addition, tumor growth delay (TGD, for 5-fold of the starting volume) was calculated. TGD was 1.58 days for 3M-011 and 6.11 days for Rx (average time to reach 5-fold tumor volume: control 6.89 (4-14) days, 3M-011 8.47 (2-16) days, Rx 13.0 (2-28) days). TGD for the combination treatment could not be calculated as tumors never reached 5-fold of their starting volume, in both monotherapy groups some tumors did not reach 5-fold of their volume during the course of the experiment.

We next tested the treatment combination in a subcutaneous model of pancreatic cancer by implanting syngeneic Panc-02 tumor fragments subcutaneously into C57Bl/6 mice. Treatment was again initiated after the establishment of local tumors. In this tumor model, the effects of the 3M-011 monotherapy were less pronounced than in CT26 tumors. Nevertheless, the combination treatment showed a statistically significant inhibition of growth of the tumors (p<0.05) and the addition of 3M-011 to radiotherapy produced a statistically significant increase in treatment efficacy over irradiation alone (p<0.05, Fig. 2B). TGD (due to the slower growth of Panc-02 tumors as compared to CT26 tumors calculated for 3-fold of the starting volume) was 3.32 days for 3M-011, 1.44 days for Rx and 6.12 days for the combined treatment (average time to 3-fold tumor volume: control 4.35 (2-11) days, 3M-11 7.67 (4-18) days, Rx 5.79 (2-16) days, 3M-011 + Rx 10.47 (2-23) days).

### Treatment with 3M-011 and radiation inhibits tumor growth and metastasis in orthotopic pancreatic cancer

In gastrointestinal tumors, the survival of patients is often limited by metastases rather than by the primary tumor itself. Subcutaneous models are mostly models for primary tumor growth. We therefore tested the radio-/immunotherapy combination in a syngeneic, orthotopic mouse model of pancreatic cancer that serves as a model for both local tumor growth and lymph node metastasis [18].

As in the subcutaneous models, we observed a significant reduction in tumor growth achieved by TLR agonist monotherapy (p<0.05). Radiation therapy was delivered to an epigastric target field and also significantly reduced the tumor volume and weight at the end of the experiments (p<0.05). The combination treatment performed significantly better than each monotherapy (p<0.01, Fig. 2C).

The tumors in this mouse model regularly metastasize to mesenteric lymph nodes of the small intestine. The metastatic activity of the primary tumor can be measured by quantification of mesenteric lymph node metastases aligned along the blood vessels. The tumors treated with 3M-011 alone showed a reduced metastatic activity, but failed to reach statistical significance. (33±24% reduction, n=8-10, n.s.). When combined with radiation therapy, this effect was significantly more pronounced (73±21% reduction as compared to radiation alone, 91±7% reduction as compared to the control group, n=8-10, p<0.05 (dual treatment vs Rx alone), p<0.0001 (dual treatment vs. control), Fig. 2D-F).

In addition to the postmortem measurements, *in vivo* FDG-PET imaging was performed on selected mice from all groups. A decline in metabolic activity in the epigastric target areas was noted in animals treated with both radiation and TLR agonist. Due to the low number of animals subjected to PET imaging, statistical analysis was not feasible (Supplemental Fig. 2).

### 3M-011 activates NK cells indirectly via monocyte-derived IL-6

As 3M-011 had minimal direct effects on tumor cells, we concluded that the antitumor effects *in vivo* were indirect in nature. Previous reports had indicated a pivotal role of NK cells in the treatment with TLR agonists [5], however, the role of other immune cell populations during treatment with TLR7/8 or together with radiation combinations remained unknown.

**Fig.3:**
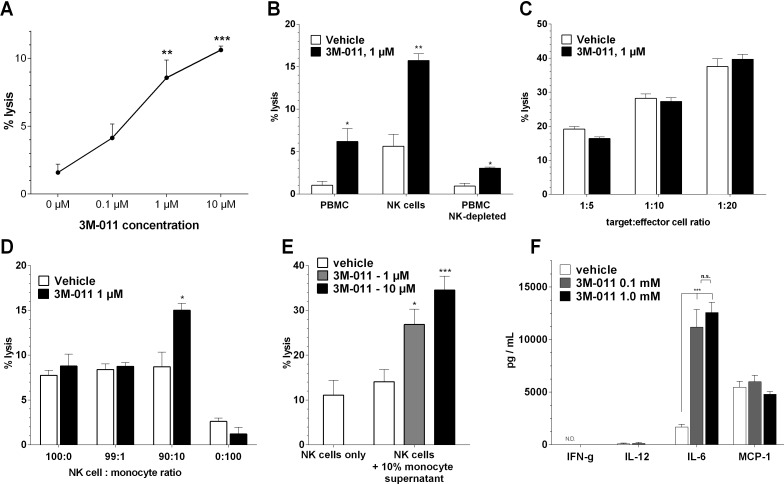
*In vitro* assays A. Dose-dependent induction of human PBMC cytotoxity by 3M-011 in an ADCC assay with HT-29 CRC cells as target cells. B. ADCC assay of human PBMC (left), MACS-enriched CD56^+^ NK cells (middle) and CD56-depleted PBMC treated with 3M-011 or vehicle. C. ADCC assay of FACS-sorted CD56^+^ NK cells in various target:effector cell ratios treated with 3M-011 or vehicle. D. ADCC assay of a mixture of FACS-sorted CD56^+^ NK cells and monocytes in various ratios, treated with 3M-011 or vehicle. E. ADCC assay of FACS-sorted CD56^+^ NK cells incubated with supernatants of monocytes treated with increasing concentrations of 3M-011. F. ELISA quantification of various cytokines in the monocyte supernatants used in (E). *All values represent mean ± SEM.*

To further investigate the tumor/immune-cell interaction during treatment with 3M-011, we first performed ^51^Cr release cytotoxicity assays on human PBMC stimulated with various doses of 3M-011. The TLR ligand dose-dependently increased the cytotoxic activity of human peripheral blood mononuclear cells (PBMC) on HT-29 CRC tumor cells (Fig. 3A). We next aimed to identify the immune cell subpopulations contained within the PBMC fraction responsible for this cytotoxic response. As previous reports indicated a pivotal role of NK cells in the effects of 3M-011 *in vivo* [5], we performed MACS isolation of CD56^+^ NK cells from PBMC and evaluated their response to the TLR ligand in cytotoxicity assays. In addition to the expectedly increased baseline cytotoxic activity compared to unselected PBMC, NK cells also showed a marked response to 3M-011. Conversely, the cytotoxic activity and response to 3M-011 of CD56-depleted PBMC was markedly reduced (Fig. 3B).

Previous reports indicated that invariant NK cells could not directly be activated by TLR7/8 ligands although they do express TLR 7 [19]. The authors showed that monocyte-derived DC are required for the activation of NK cells by a TLR7/8 agonist. To test this hypothesis with 3M-011, we first isolated highly purified (>98% purity as opposed to ~90% after MACS purification) CD56^+^ NK cells by FACS sorting. These cells were subsequently used in the cytotoxicity assays (Fig. 3C) and exhibited increasing target cell lysis with increasing effector:target cell ratios. However, the addition of the TLR ligand to the culture medium did not cause any significant increase in cytotoxicity. As NK cells with relatively low purity (~90%) responded to 3M-011 stimulation whereas highly purified (>98%) NK cells did not, we hypothesized that co-stimulation by a subpopulation in the contaminating cell fraction of the MACS-purified NK cells must be required for NK cells to respond to 3M-011. As previous reports indicated that this contaminating fraction may be cells of the monocytic lineage [19], we artificially contaminated FACS-sorted (i.e., highly purified) NK cells with PBMC-derived monocytes in various ratios and tested the cell mixture for its antitumor cytotoxicity. Fig. 3D shows that a contaminating monocyte fraction of 10% was required to elicit a cytotoxic response to 3M-011.

To test whether this effect was caused by direct cell-cell interaction or an indirect paracrine effect, we incubated monocytes with 3M-011, collected the supernatants und subsequently incubated FACS-sorted NK cells with the monocyte supernatants. Untreated monocyte supernatants were not able to significantly increase the NK cell cytotoxity. However, when pre-treated with 3M-011, the monocyte supernatants dose-dependently stimulated NK cell toxicity (Fig. 3E). ELISA analysis of the monocyte supernatants revealed a marked increase in IL-6 levels upon TLR stimulation, which may explain the increased NK cell activity [20,21].

### Tumor-specific cytotoxic T cells are the mediators of the effects of combined radio-immunotherapy

To determine the main effectors of the *in vivo* antitumor activity, we next depleted wild-type C57Bl/6 mice of either NK cells or CD8 T cells by monoclonal antibody injection (PK136 for NK cells, 53-6.72 for CD8 T cells), as described previously [22,23]. The efficacy and durability of the depletion was repeatedly verified by FACS analyses of mouse blood samples throughout the experiment (Supplemental Fig. 3).

**Fig.4:**
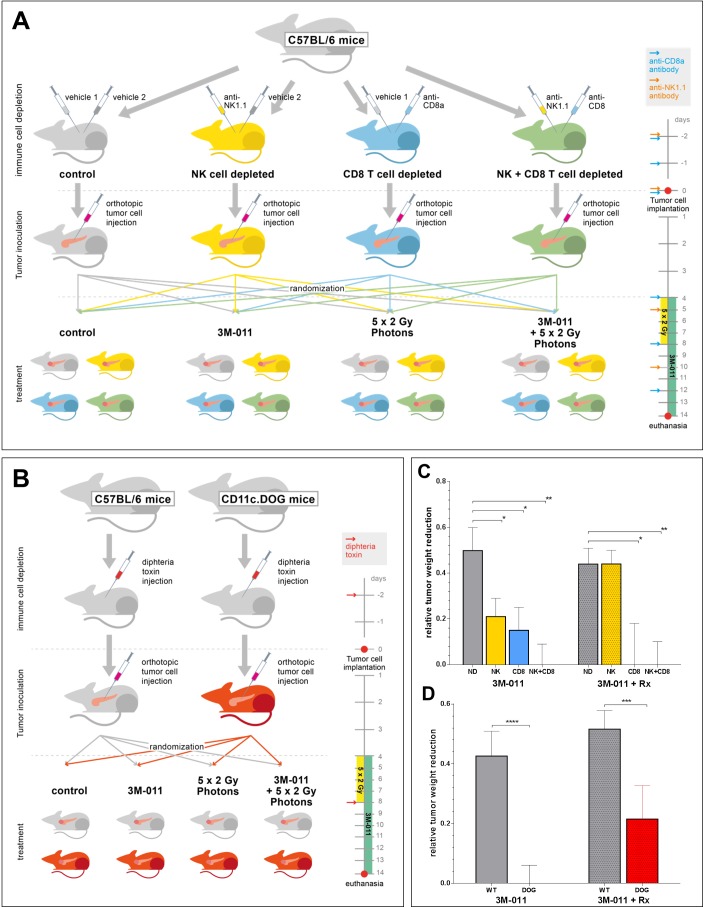
A+B: Experimental set-up of the *in vivo-*depletion experiments A. NK + CD8 T cell depletion; B. Dendritic cell depletion. Injection algorithms for immune cell depletion are given in the timelines on the right hand sides of the sketches. C+D: Relative tumor weights at necropsy 11 days after orthotopic inoculation of Panc-02 PDAC cells in immune cell-depleted mice. C. Mice treated with 3M-011 (left 4 bars) or 3M-011 + 5× 2 Gy photons (right 4 bars) and depleted of either NK cells (NK), CD8 T cells (CD8), or both (NK+CD8). All bars indicate relative values compared to their 3M-011-naïve controls (left 4 bars show relative values as compared to untreated mice; right 4 bars as compared to the Rx monotherapy group). D. Mice (CD11c.DOG / C57Bl/6) treated with 3M-011 (left 2 bars) or 3M-011 + 5× 2 Gy photons (right 2 bars) and repeated diphtheria toxin injections. Again, all bars are compared to their 3M-011 native controls (left 2 bars: compared to untreated mice; right 2 bars: compared to the Rx monotherapy group). *CD8, CD8 T cell depleted; DOG, CD11c-DOG mice; ND, non-depleted; NK, NK cell depleted; Rx, radiation therapy; WT, C57Bl/6 wild-type mice. All values represent mean ± SEM.*

Healthy mice were divided into 4 groups (non-depleted (ND), NK cell depleted (NK), CD8 T cell depleted (CD8) and combined depletion (NK + CD8)) and pre-treated with the depletion mAbs according to protocol (Fig. 4A). After the inoculation of orthotopic pancreatic tumors, each group was then randomly divided into 4 subgroups (vehicle, 3M-011, Rx (5×2 Gy), 3M-011 + Rx) and treated as indicated. Radiation therapy was again delivered in five 2 Gy fractions to an epigastric target field. Tumor inoculation and data collection at the end of the experiment were carried out following the protocol of the orthotopic pancreatic cancer model.

In mice treated with either vehicle or 3M-011 monotherapy, the effects of 3M-011 appeared to be based on the presence of both NK and CD8 T cells (Fig. 4C). In the groups depleted of either cell population, the efficacy of 3M-011 was reduced by about 50-70% compared to non-depleted (ND) mice (p<0.05 for both comparisons). In the double-depleted group, 3M-011 had no measurable antitumor effect.

Interestingly, the anti-tumor effects of the combination of TLR7/8 agonist and radiation appeared to be entirely mediated by CD8 T cells. The efficacy of 3M-011 treatment in the combination group was not affected by NK cell depletion, as the results in the dual depleted group were identical to the results of the CD8 depleted group. In mice depleted of CD8 T cells, however, the addition of 3M-011 to radiation therapy produced no additional effects.

### CD11c^+^ dendritic cell-mediated NK and T-cell priming is required for the effects of the treatment combination

Since both CD8 T cells and NK cells are dependent on previous activation by antigen-presenting cells such as DC, we aimed to investigate the role of DC by selectively depleting these cells in tumor-bearing and treated mice. To this end, we used CD11c.DOG mice, which allow selective and long-term depletion of CD11c^+^ DC without otherwise affecting the animals [24]. CD11c.DOG are transgenic mice, expressing the diphtheria toxin receptor (DTR) under control of the CD11c promoter. All CD11c^+^ cells in these mice express DTR and can therefore be selectively ablated by systemic administration of diphtheria toxin (DT). We used the orthotopic Panc-02 model in C57Bl/6 CD11c.DOG mice. To exclude effects of DT on tumor cells, we used wild-type C57Bl/6 mice as controls and treated them with DT in the same manner as the CD11c.DOG mice. Each group of mice (C57Bl/6, CD11c.DOG) was then divided in to the 4 treatment groups (vehicle, 3M-011, Rx, 3M-011 + Rx) and treated as indicated (Fig. 4B). The radiation was again targeted to the epigastrium including the entire pancreas.

Tumor growth and metastasis in DT treated wild-type mice was similar to non-DT-treated mice. Tumor response to both treatment modalities was also consistent with previous results. In CD11c.DOG mice, however, DC ablation led to a near complete abolition of the effects of 3M-011 (Fig. 4D). The effects of radiation alone were also reduced by about 50% (data not shown). Similarly, DC depletion markedly reduced the anti-tumor effects in the combination group of 3M-011 and radiation.

The strong effects of the treatment combination in the previous experiments were indicative of a therapeutically boosted specific immune response. Therefore, we investigated if the treatment combination increased the number of tumor-specific T lymphocytes *in vivo.* To accomplish this, subcutaneous CT26 tumors were induced in BALB/c mice, treated according to protocol and then T cells were extracted from the spleens to perform ELISPOT assays. The antigens used in this assay were AH1 (a tumor antigen highly expressed in the CT26 cell line) and OVA (control peptide). The ELISPOT assay revealed an increase in CT26-specific T-cells by 3M-011 which was even more augmented by additional radiation (Supplemental Fig. 4).

### 3M-011 shifts the intratumoral and systemic cytokine equilibrium to a pro-inflammatory state

To evaluate the effects of the TLR ligand on the intratumoral cytokine profile, tumor tissue of subcutaneous tumors were analysed from mice treated with 3M-011 and ionizing radiation. Changes in the intratumoral cytokine levels of MCP-1, IL-6, INF-γ and TNF-α were measured with the BD Cytometric Bead Array.

The intratumoral levels of all four cytokines were increased by the TLR agonist. For MCP-1, INF-γ and TNF-α, the increase induced by 3M-011 was statistically significant (MCP-1, p<0.0001; INF-γ, p=0.0098; TNF-α, p=0.0024), but the IL-6 increase failed to reach statistical significance (p=0.1451). When combined with ionizing radiation, the intratumoral levels of all cytokines except MCP-1 were further increased (Supplemental Fig. 5A).

Consistent with the increased cytokine levels within the tumors, treatment induced more pronounced systemic cytokine responses. Interestingly, for most cytokines, the systemic increase caused by dual treatment was smaller than the increase caused by 3M-011 monotherapy (Supplemental Fig. 5B). Of note, while INF-γ and TNF-α were not significantly affected by DC depletion, the systemic levels of MCP-1 and IL-6 were significantly reduced after DT administration in CD11c.DOG mice (data not shown).

## DISCUSSION

Here we show that the TLR7/8 agonist 3M-011 is a potent adjuvant to radiotherapy in syngeneic and orthotopic mouse models of colorectal and pancreatic cancer that resulted in marked local and systemic antitumor activity. The rationale of the therapeutic combination was that radiotherapy would provide tumor antigens, while the TLR7/8 agonist would stimulate antigen-presenting cells to process and present these antigens in order to induce and augment an immune response to the tumor (Fig. 1). Mechanistically, 3M-011 activation of APC was essential for NK activation, and both NK and CD8 T cells contributed to antitumor responses to 3M-011 as a single agent. In combination with irradiation, however, CD8 effector T cells dominated the antitumor responses. Nevertheless, DC still had a significant impact on the antitumor response to 3M-011 plus irradiation, since their depletion significantly decreased treatment efficacy. In addition, 3M-011 in combination with irradiation substantially increased intratumoral INF-γ, which in turn may contribute to activation of CD8 effector cells. These mechanisms appear to act in concert to induce a strong anti-tumor immune reaction, which was markedly enhanced by the TLR7/8 agonist over the direct effects of radiation alone.

Tumor cell expression of TLR in carcinomas have been associated with increased growth and invasiveness upon TLR activation [17]. However, 3M-011 did not stimulate the murine or human PDAC and CRC cell lines *in vitro*, nor did it enhance tumor growth in any of the mouse models. There may be several possible explanations for the differences observed between our study and that of Ochi et al. One reason for the difference might be the different TLR agonists used. Ochi et al. used ssRNA40, *E. coli* RNA, or Adenine analogs as TLR agonists, which may activate additional signaling pathways than just TLR7/8 as compared to the here used specific TLR7/8 agonist. Another possible explanation may be tumor microenvironment. Although TLR7 was observed in PDAC tumor cells by Ochi et al., they reported that the effects of TLR7 activation on the tumor cells was secondary to TLR7 activation in the surrounding inflammatory cells (28). Subcutaneous tumors (Fig. 2 A,B) are likely to have a different microenvironment than a tumor that develops naturally. Even orthotopic homografts from established cell lines might have a different microenvironment, or might respond differently to their surroundings. Thus, either a different microenvironment or response to the microenvironment might explain the apparent discrepancy between our results and those previously published.

In the subcutaneous tumor models combining 3M-011 with radiotherapy, we observed a significant number of complete remissions despite the relatively low dose of radiation. These effects were confirmed in the orthotopic model of pancreatic cancer, where both primary tumor growth and metastasis were dramatically reduced. In order to characterize the immune cells responsible for these beneficial effects, a series of *in vitro* and *in* vivo assays were performed. Using *in vitro* assays, we were able to show that NK cells were involved in mediating the effects of 3M-011, but required co-stimulation by monocytic cells, which produced significant amounts of IL-6 upon 3M-011 stimulation. This finding is consistent with previous reports indicating low TLR7/8 expression in NK cells and indirect stimulatory effects of TLR agonists on them [19,25].

By *in vivo* depletion of NK and/or CD8 T cells we showed that 3M-011 monotherapy was dependent on both NK cells and CD8 T cells, both of which contributed approximately 50% of the antitumor effect. Interestingly, when combined with ionizing radiation, the role of CD8 T cells became dominant while the role of NK cells appeared to be negligible. We speculate that this might be the result of an “intrinsic vaccination” effect, which may also explain the beneficial interaction of radiation and TLR stimulation. While the TLR agonist can activate cells of both the adaptive and innate immune systems, its main target cells are DC, which phagocytose, process and present antigens to T cells. Such antigens are released in abundance by radiation-induced cell death, which explains the synergistic effects of the dual treatment. Of note, the tumor sizes in the untreated control groups were not affected by either NK or CD8 T cell depletion, indicating effective immune escape mechanisms of the untreated tumor homografts.

To demonstrate the importance of DC in therapy-induced T cell activation, we used CD11c-DTR transgenic mice. Depletion of DC eliminated the efficacy of 3M-011 monotherapy and significantly reduced the effects of 3M-011 + radiation therapy. The therapy-induced increase of tumor-specific T cells was also demonstrated in ELISPOT assays, which was consistent with data from other groups reporting increased specific immune responses to antitumor vaccination when combined with a TLR7/8 agonist [26].

Cytokine responses within the tumor were more subtle than those observed in plasma, but only within the tumor could induction of cytokines be observed in response to irradiation monotherapy. In contrast, only 3M-011 induced cytokine elevations in plasma. The dramatic cytokine response (“cytokine storm”) caused by systemic 3M-011 administration was well tolerated by the animals, but such responses in humans might cause side effects such as flu-like symptoms. Depending on the severity of these side effects, systemic induction of cytokines could result in a dose-limiting toxicity that might require dose reductions or discontinuation of therapy [27]. These considerations suggest that a locally applied TLR7/8 agonist might be preferable.

Imiquimod (Aldara cream) is a topically administered TLR agonist, and the only one that has been clinically approved to date. Imiquimod shows significant activity in many skin conditions including basal cell carcinoma, actinic keratosis (a precancerous form of squamous cell carcinoma [28]) and, off label, melanoma [29]. While topical TLR agonists and radiation combinations have shown promise [30], gastrointestinal tumors are not accessible to topical treatment and require other ways of administration. For the treatment of CRC and PDAC, intratumoral administration (e.g., endoscopically or interventionally) could be a feasible way of administration. Such topical administration would be expected to activate the immune cells in contact with tumor antigens while avoiding excessive systemic cytokine release. In patients with metastatic cancer, an interesting question will be whether local treatment of a single metastatic lesion can induce a systemic immune response. For example, distant metastases in the liver or the skin may be easily accessible to local or even topical treatment. The induction of a systemic immune reaction by treating a single, easily accessible lesion would potentially greatly benefit patients with a yet dismal prognosis and has anecdotally been reported in other tumor entities before [31].

Two recent studies recently published that used a similar approach of combining radiation and another TLR7/8 agonist in a lymphoma [32] and another GI and a fibrosarcoma tumor model [33] are in general agreement with our data, thus independently confirming the combination benefits and supporting the high translational relevance of this therapeutic strategy for a wide range of tumors. Besides demonstrating that this approach may be applicable to additional types of tumors, our data provide conclusive evidence that it is CD11c^+^ DC that mediate the therapeutic effects via NK and CD8 T cell activation and priming.

In summary, the data presented here clearly show a marked antitumoral activity of 3M-011 combined with ionizing radiation. The effects are mediated by NK and, predominantly, cytotoxic T cells that require activation by DC, which are the main target of the TLR7/8 agonists. These observations suggest that further investigation is warranted.

## MATERIAL AND METHODS

### Cell lines

The human pancreatic adenocarcinoma cell lines Panc-1 and BxPC3, the human colorectal carcinoma cell lines HT29 and HCT-116, and the murine colorectal carcinoma cell line CT26 were purchased from the American Type Culture Collection (ATCC) in 2008. The murine pancreatic carcinoma cell line Panc-02 was generously provided by P. Carmeliet (VIB Vesalius research Center, Leuven, Belgium) in 2010. Panc-1, BxPC3, HT29 and Panc-02 were propagated in RPMI (PAA)) supplemented with 10% heat-inactivated fetal bovine serum, 100 U/mL penicillin, and 100 μg/mL streptomycin. HCT-116 and CT26 were propagated in DMEM (PAA) supplemented with 10% heat-inactivated fetal bovine serum, 100 U/mL penicillin, and 100 μg/mL streptomycin. All cell lines were regularly tested for contamination and authenticated by the DKFZ Genome and Proteome Core Facility via SNP profiling. The last authentication was done in 2012, shortly before the experiments with cell lines were completed.

### Proliferation and apoptosis assays

Cell lines were cultured in a final volume of 100 μl/well culture medium in 96 well flat bottom cell culture microplates and incubated overnight. Different starting cell concentrations were assessed. (0/well, 250/well, 500/well, 1000/well). Cells were then treated with 0.1, 1.0 and 10 μM 3M-011 or sodium acetate buffer as a control for 96 hours. Then 100 μl/well WST-1 reagent was added. Cells were incubated in a 37 °C incubator maintained at 5% CO_2_ for 3 hours, then were shaken thoroughly for 1 min. Absorbance of the samples was measured using a microplate reader at 480 nm. All experiments were done in quadruplicates.

### Mouse models

BALB/c and C57Bl/6 mice were obtained from Charles River Laboratories. CD11c.DOG mice were kindly provided by Dr. Günter Hämmerling, DKFZ, Germany. All mice were maintained under specific pathogen-free conditions with a 12hr light/dark cycle and had *ad libitum* access to standard laboratory diet and water. All animal experiments were evaluated and permitted by both institutional and governmental animal welfare committees (Regierungspräsidium Karlsruhe) and performed in strict accordance to FELASA and GV-SOLAS guidelines.

Subcutaneous tumor models: 1× 10^5^ tumor cells (CT26 or Panc-02) were injected s.c. into syngeneic animals (BALB/c or C57Bl/6). Once the resulting tumors reached a size of 10-12 mm in largest diameter, the tumors were excised and 1×1 mm fragments were implanted subcutaneously into the right flank of BALB/c or C57Bl/6 animals. Tumor volumes were subsequently measured every other day with a caliper, the formula:

$V=\;\frac{dx\;dx\;D}{2}$, where d is the minor tumor axis and D is the major tumor axis, was used to estimate the tumor volume. Mice were euthanized when tumors reached 20 mm in largest diameter or suffering of the animals became apparent.

Orthotopic PDAC model: 1× 10^6^ Panc02 tumor cells were surgically injected into the head of the pancreas. Mice were euthanized after 11 days or when suffering of the animals became apparent.

### Radiation therapy

Irradiation was delivered in 2 Gray fractions on 5 subsequent days on a Siemens Gammatron unless indicated otherwise. Tumor-surrounding tissue was covered using lead shields.

### 3M-011 treatment

3M-011 was dissolved in 0,1 M sodium acetate buffer at pH=5.2 and mice were injected i.p. with 200 μl of 3M-011 at dose of 5mg/kg body weight every other day until termination of the experiment. Control group mice received 200 μl of sodium acetate buffer in the same volume and regimen.

For subcutaneous tumors, treatment was initiated when the tumors reached an average diameter of 6 mm. In the orthotopic mouse model treatment was initiated on day 2 after tumor cell inoculation.

### Positron emission tomography (PET)

The animals were fasted for 4 h prior to PET and kept in an inhalation narcosis with sevorane (0.5 volume-%) and oxygen (flow = 500 ml/min) during the PET examination. The plasma glucose level of the animals was determined using a blood glucose sensor electrode (MediSense) with glucose levels between 100 and 135 mg/dl. A transmission scan was done for 10 minutes prior to tracer administration with two rotating germanium pin sources to obtain cross sections for attenuation correction. The injected activity was 8-9.9 MBq and adjusted according to the weight of the animal which ranged from 14 g to 25 g. PET data were acquired for 20 minutes at 40 to 60 minutes p.i. on an Siemens Inveon scanner (Siemens) using a matrix of 256 × 256 (pixel size 0.3882 × 0.3882 × 0.796 mm). Thereafter, images were reconstructed iteratively using the space alternating generalized expectation maximization method (SAGE, 16 subsets, 4 iterations) applying median root prior correction and were converted to standardized uptake value (SUV) images on the basis of the formula

$$SUV=\frac{\text{tissue concentration}(\text{Bq}/\text{g})}{\text{injected dose }[\text{Bq}]/\text{body weight }[\text{g}]}.$$

### *In vivo* depletion of NK cells, cytotoxic T-lymphocytes and dendritic cells

Depletion of NK cells was performed by i.p. injection of 300 μg anti-NK1.1 mAb PK136 (BioXCell) / mouse as described before [22]. Antibody treatment was done 2 d prior to tumor cell inoculation and on days 0,5, and 10 of the experiment (with 0 being the day of tumor cell inoculation). For depletion of cytotoxic T cells, mice were injected i.p. with 200 μg anti-CD8 mAb 53.6-72 (BioXCell) 2 days and 1 day before infection and subsequently on days 0, 4, and 8 as described before [23]. Effectiveness of depletion confirmed in blood on days 0, 4, and 8 by flow cytometry. >98% depletion of CD8 T cells and >95% depletion of NK cells were achieved, respectively. All other groups were treated with the according isotype control antibodies (BioXCell, Fig. 4).

For depletion of DC, CD11c.DOG mice were treated with 8 ng/g body weight of diphtheria toxin (DT) i.p. on day −2, 0, 4, 8, 12, and 16 (with 0 being the day of tumor cell inoculation). CD11c^+^ cell depletion efficacy was assessed using flow cytometry on days 0, 4, and 8.

### Natural killer cell cytotoxicity assay (NKCC)

The bioassay for NKCC was performed using whole blood within 8 hours of collection in a chromium release assay as previously described [34]. The human colorectal carcinoma cell line HT29 was used as target. NK cells were incubated for 16h with indicated concentrations of 3M-011 or cell supernatant prior to NKCC assay. The assay was done in triplicates with 4 hours of incubation time and the relative cytotoxic activity was calculated as follows:

$$\frac{[\%]\text{ Chromium release in test }-\;[\%]\text{ Chromium spontaneous release}}{[\%]\text{ Chromium release in max.lysis positive control}-[\%]\text{ Chromium spontaneous release}}$$

### Cell Isolation and Sorting

Peripheral blood mononuclear cells (PBMC) were isolated from freshly obtained blood from healthy donors by Ficoll density gradient centrifugation (GE Healthcare, USA) as described previously [35].

Cell sorting was performed on a FACS Aria II sorting system (BD Biosciences) using MEM-188-APC for CD56, 3G8-Cy5.5 for CD16, and hCD14-FITC for CD14 (all from Biolegend).

### Flow cytometry

Samples were analyzed on a standard BD FACS Calibur flow cytometer (BD Biosciences). Samples were stained according to the manufacturer's protocols for staining of surface antigens in FACS buffer (PBS, 1% fetal calf serum). Samples were analyzed as indicated using the following antibodies: 3G8-Cy5.5 for CD16, MEM-188-APC for CD56, hCD14-FITC for CD14, OKT3-FITC for CD3, PK136-Alexa Fluor 488 for NK1.1, 53-6.7-Alexa Fluor 647 for CD8a (all from Biolegend). Samples were acquired for 3 to 4-colour analysis and analyzed using FlowJo 7.5 software (TreeStar Inc). A total of 500,000–1,200,000 cells were acquired.

### Cytokine quantification in mouse serum and tumor lysates

Serum: At day 7 after initiation of the treatment, blood was withdrawn from tumor-bearing mice by puncture of the retrobulbar venous plexus, supplemented with Complete Protease Inhibitor (Roche) and allowed to clot on ice for 30 minutes. The blood was then centrifuged and the serum supernatant obtained.

Tumor tissue lysates: To obtain tumor lysates, tumors were macrodissected and 50 μg of tumor tissue were lysed in 1 ml M-PER Mammalian Protein Extraction Reagent (Thermo Scientific) supplemented with Complete Protease Inhibitor (Roche) in a TissueLyser II (Qiagen) according to the manufacturers' instructions. After centrifugation, the supernatant was obtained and used for cytokine quantification.

ELISA: The following ELISA kits where used accordingly to manufacturers' protocols: LEGEND MAX Human INF-γ ELISA Kit, LEGEND MAX Human IL-6 ELISA Kit, LEGEND MAX Human IL-12 (p70) ELISA Kit, LEGEND MAXHuman IL-15 ELISA Kit, LEGEND MAXHuman MCP-1/CCL2 ELISA Kit, LEGEND MAX Human TNF-α ELISA Kit (all from R&D) and Human INF-α ELISA Kit, Human IL-18/IL-1F4 ELISA (all from Biolegend).

BD Cytometric Bead Arrays: Proinflammatory murine cytokines (INF-γ, TNF-α, MCP-1, IL-6, IL-10 IL-12p70) were quantified simultaneously using the Mouse Inflammation Cytokine Cytometric Bead Array Kit and CBA software (BD Pharmingen). The kit was used according to manufacturer's instructions on a FACSCalibur flow cytometer (BD Biosciences, San Jose, CA) on mouse sera and tumor lysates. To obtain tumor lysates, tumors were macrodissected and 50 μg of tumor tissue were lysed in 1 ml M-PER Mammalian Protein Extraction Reagent (Thermo Scientific) supplemented with Complete Protease Inhibitor (Roche) in a TissueLyser II (Qiagen) according to the manufacturers' instructions.

### ELISPOT

To determine systemic tumor-reactive T cell immunity, we isolated T cells from peripheral blood and bone marrow of CT26-bearing BALB/C mice treated with 3M-011 or control. DC were derived from non-tumor-bearing BALB/C mice. We used defined synthetic polypeptides of the tumor-associated tumor antigen AH1 to pulse the DC as described previously [36]. Spot-forming cells were quantified using the KS ELISPOT reader (Zeiss) and CTL ImmunoSpot Analyzer and ImmunoSpot software (Cellular Technology). Spots were measured in the presence of DC pulsed with AH-1 (endogenous tumor antigen on CT-26 tumor cells). Spots measured in the presence of DC pulsed with Ovalbumin where considered as nonspecific background controls. All peptides where obtained from Proimmune, UK. All tests were done in triplicates.

### Statistical methods

Statistical analysis was achieved by two-tailed student's t-test. All plotted values represent mean ± SEM. p<0.05 was considered statistically significant. Graphpad Prism 6 (Graphpad Software Inc.) was used for statistical analyses and data plotting. Statistically significant values are indicated by asterisks (key: * p<0.05; ** p<0.01; *** p<0.001; **** p<0.0001).

## SUPPLEMENTARY MATERIAL FIGURES



## Abbreviations

APC: antigen-presenting cell
CRC: colorectal cancer
DC: dendritic cells
DT(R): diphtheria toxin (receptor)
IL: interleukin
INF: interferon
NK cells: natural killer cells
OVA: ovalbumin
PBMC: peripheral blood mononuclear cells
PDAC: pancreatic ductal adenocarcinoma
ND: non-depleted
Rx: radiation
TGD: tumor growth delay
TLR: Toll-like receptor
WT: wild-type

## References

[1] Weitz J, Koch M, Debus J, Höhler T, Galle PR, Büchler MW (2005). Colorectal cancer. Lancet.

[2] Siegel R, Naishadham D, Jemal A (2013). Cancer statistics, 2013. CA Cancer J Clin.

[3] Bath-Hextall F, Ozolins M, Armstrong SJ, Colver GB, Perkins W, Miller PSJ, Williams HC (2014). Surgery versus Imiquimod for Nodular Superficial basal cell carcinoma (SINS) study group. Surgical excision versus imiquimod 5% cream for nodular and superficial basal-cell carcinoma (SINS): a multicentre, non-inferiority, randomised controlled trial. Lancet Oncol.

[4] Akira S, Takeda K, Kaisho T (2001). Toll-like receptors: critical proteins linking innate and acquired immunity. Nat Immunol.

[5] Dumitru CD, Antonysamy MA, Gorski KS, Johnson DD, Reddy LG, Lutterman JL, Piri MM, Proksch J, McGurran SM, Egging EA, Cochran FR, Lipson KE, Tomai MA (2009). NK1. 1+ cells mediate the antitumor effects of a dual Toll-like receptor 7/8 agonist in the disseminated B16-F10 melanoma model. Cancer Immunol Immunother CII.

[6] Dumitru CD, Antonysamy MA, Tomai MA, Lipson KE (2010). Potentiation of the anti-tumor effects of imidazoquinoline immune response modifiers by cyclophosphamide. Cancer Biol Ther.

[7] Seremet T, Brasseur F, Coulie PG (2011). Tumor-specific antigens and immunologic adjuvants in cancer immunotherapy. Cancer J Sudbury Mass.

[8] Baxevanis CN, Voutsas IF, Tsitsilonis OE (2013). Toll-like receptor agonists: current status and future perspective on their utility as adjuvants in improving anticancer vaccination strategies. Immunotherapy.

[9] Steinman RM, Banchereau J (2007). Taking dendritic cells into medicine. Nature.

[10] Beignon A-S, McKenna K, Skoberne M, Manches O, DaSilva I, Kavanagh DG, Larsson M, Gorelick RJ, Lifson JD, Bhardwaj N (2005). Endocytosis of HIV-1 activates plasmacytoid dendritic cells via Toll-like receptor-viral RNA interactions. J Clin Invest.

[11] Formenti SC, Demaria S (2013). Combining radiotherapy and cancer immunotherapy: a paradigm shift. J Natl Cancer Inst.

[12] Kalbasi A, June CH, Haas N, Vapiwala N (2013). Radiation and immunotherapy: a synergistic combination. J Clin Invest.

[13] Finkelstein SE, Fishman M (2012). Clinical opportunities in combining immunotherapy with radiation therapy. Front Oncol.

[14] Rödel F, Frey B, Multhoff G, Gaipl U (2013). Contribution of the immune system to bystander and non-targeted effects of ionizing radiation. Cancer Lett.

[15] Klug F, Prakash H, Huber PE, Seibel T, Bender N, Halama N, Pfirschke C, Voss RH, Timke C, Umansky L, Klapproth K, Schäkel K, Garbi N (2013). Low-dose irradiation programs macrophage differentiation to an iNOS+/M1 phenotype that orchestrates effective T cell immunotherapy. Cancer Cell.

[16] Garbi N, Arnold B, Gordon S, Hämmerling GJ, Ganss R (2004). CpG motifs as proinflammatory factors render autochthonous tumors permissive for infiltration and destruction. J Immunol Baltim Md 1950.

[17] Ochi A, Graffeo CS, Zambirinis CP, Rehman A, Hackman M, Fallon N, Barilla RM, Henning JR, Jamal M, Rao R, Greco S, Deutsch M, Medina-Zea MV (2012). Toll-like receptor 7 regulates pancreatic carcinogenesis in mice and humans. J Clin Invest.

[18] Loges S, Schmidt T, Tjwa M, van Geyte K, Lievens D, Lutgens E, Vanhoutte D, Borgel D, Plaisance S, Hoylaerts M, Luttun A, Dewerchin M, Jonckx B (2010). Malignant cells fuel tumor growth by educating infiltrating leukocytes to produce the mitogen Gas6. Blood.

[19] Moreno M, Mol BM, von Mensdorff-Pouilly S, Verheijen RHM, de Jong EC, von Blomberg BME, van den Eertwegh AJM, Scheper RJ, Bontkes HJ (2009). Differential indirect activation of human invariant natural killer T cells by Toll-like receptor agonists. Immunotherapy.

[20] Luger TA, Krutmann J, Kirnbauer R, Urbanski A, Schwarz T, Klappacher G, Köck A, Micksche M, Malejczyk J, Schauer E (1989). IFN-beta 2/IL-6 augments the activity of human natural killer cells. J Immunol Baltim Md 1950.

[21] Luger TA, Schwarz T, Krutmann J, Kirnbauer R, Neuner P, Köck A, Urbanski A, Borth W, Schauer E (1989). Interleukin-6 is produced by epidermal cells and plays an important role in the activation of human T-lymphocytes and natural killer cells. Ann N Y Acad Sci.

[22] Wang M, Ellison CA, Gartner JG, HayGlass KT (1998). Natural killer cell depletion fails to influence initial CD4 T cell commitment *in vivo* in exogenous antigen-stimulated cytokine and antibody responses. J Immunol Baltim Md 1950.

[23] Kruisbeek AM, John E, Coligan Al (2001). *In vivo* depletion of CD4- and CD8-specific T cells. Curr Protoc Immunol.

[24] Hochweller K, Striegler J, Hämmerling GJ, Garbi N (2008). A novel CD11c. DTR transgenic mouse for depletion of dendritic cells reveals their requirement for homeostatic proliferation of natural killer cells. Eur J Immunol.

[25] Gorski KS, Waller EL, Bjornton-Severson J, Hanten JA, Riter CL, Kieper WC, Gorden KB, Miller JS, Vasilakos JP, Tomai MA, Alkan SS (2006). Distinct indirect pathways govern human NK-cell activation by TLR-7 and TLR-8 agonists. Int Immunol.

[26] Shackleton M, Davis ID, Hopkins W, Jackson H, Dimopoulos N, Tai T, Chen Q, Parente P, Jefford M, Masterman K-A, Caron D, Chen W, Maraskovsky E (2004). The impact of imiquimod, a Toll-like receptor-7 ligand (TLR7L), on the immunogenicity of melanoma peptide vaccination with adjuvant Flt3 ligand. Cancer Immun.

[27] Horscroft NJ, Pryde DC, Bright H (2012). Antiviral applications of Toll-like receptor agonists. J Antimicrob Chemother.

[28] Kerr C (2002). “Rub on” treatment for basal-cell carcinoma. Lancet Oncol.

[29] Moon SD, Spencer JM (2013). Clearance of invasive melanoma with topical imiquimod. J Drugs Dermatol JDD.

[30] Dewan MZ, Vanpouille-Box C, Kawashima N, DiNapoli S, Babb JS, Formenti SC, Adams S, Demaria S (2012). Synergy of topical toll-like receptor 7 agonist with radiation and low-dose cyclophosphamide in a mouse model of cutaneous breast cancer. Clin Cancer Res Off J Am Assoc Cancer Res.

[31] Naylor MF, Chen WR, Teague TK, Perry LA, Nordquist RE (2006). In situ photoimmunotherapy: a tumour-directed treatment for melanoma. Br J Dermatol.

[32] Dovedi SJ, Melis MHM, Wilkinson RW, Adlard AL, Stratford IJ, Honeychurch J, Illidge TM (2013). Systemic delivery of a TLR7 agonist in combination with radiation primes durable antitumor immune responses in mouse models of lymphoma. Blood.

[33] Adlard AL, Dovedi SJ, Telfer BA, Koga-Yamakawa E, Pollard C, Honeychurch J, Illidge TM, Murata M, Robinson DT, Jewsbury PJ, Wilkinson RW, Stratford IJ (2014). A novel systemically administered toll-like receptor 7 agonist potentiates the effect of ionizing radiation in murine solid tumor models. Int J Cancer J Int Cancer.

[34] Kiessling R, Klein E, Wigzell H (1975). “Natural” killer cells in the mouse. I. Cytotoxic cells with specificity for mouse Moloney leukemia cells. Specificity and distribution according to genotype. Eur J Immunol.

[35] Hoechst B, Ormandy LA, Ballmaier M, Lehner F, Krüger C, Manns MP, Greten TF, Korangy F (2008). A new population of myeloid-derived suppressor cells in hepatocellular carcinoma patients induces CD4(+)CD25(+)Foxp3(+) T cells. Gastroenterology.

[36] Koch M, Beckhove P, Op den Winkel J, Autenrieth D, Wagner P, Nummer D, Specht S, Antolovic D, Galindo L, Schmitz-Winnenthal FH, Schirrmacher V, Büchler MW, Weitz J (2006). Tumor infiltrating T lymphocytes in colorectal cancer: Tumor-selective activation and cytotoxic activity in situ. Ann Surg.

