# Supplementary material for: Radiotherapy combined with TLR7/8 activation induces strong immune responses against gastrointestinal tumors
Source: Oncotarget. 2014 Dec 31;6(7):4663–76. doi: 10.18632/oncotarget.3081 (PMC4467106; doi:10.18632/oncotarget.3081)
Supplement: Supplementary file 1 [file oncotarget-06-4663-s001.pdf]

Radiotherapy combined with TLR7/8 activation induces strong immune responses against gastrointestinal tumors

Supplementary Material

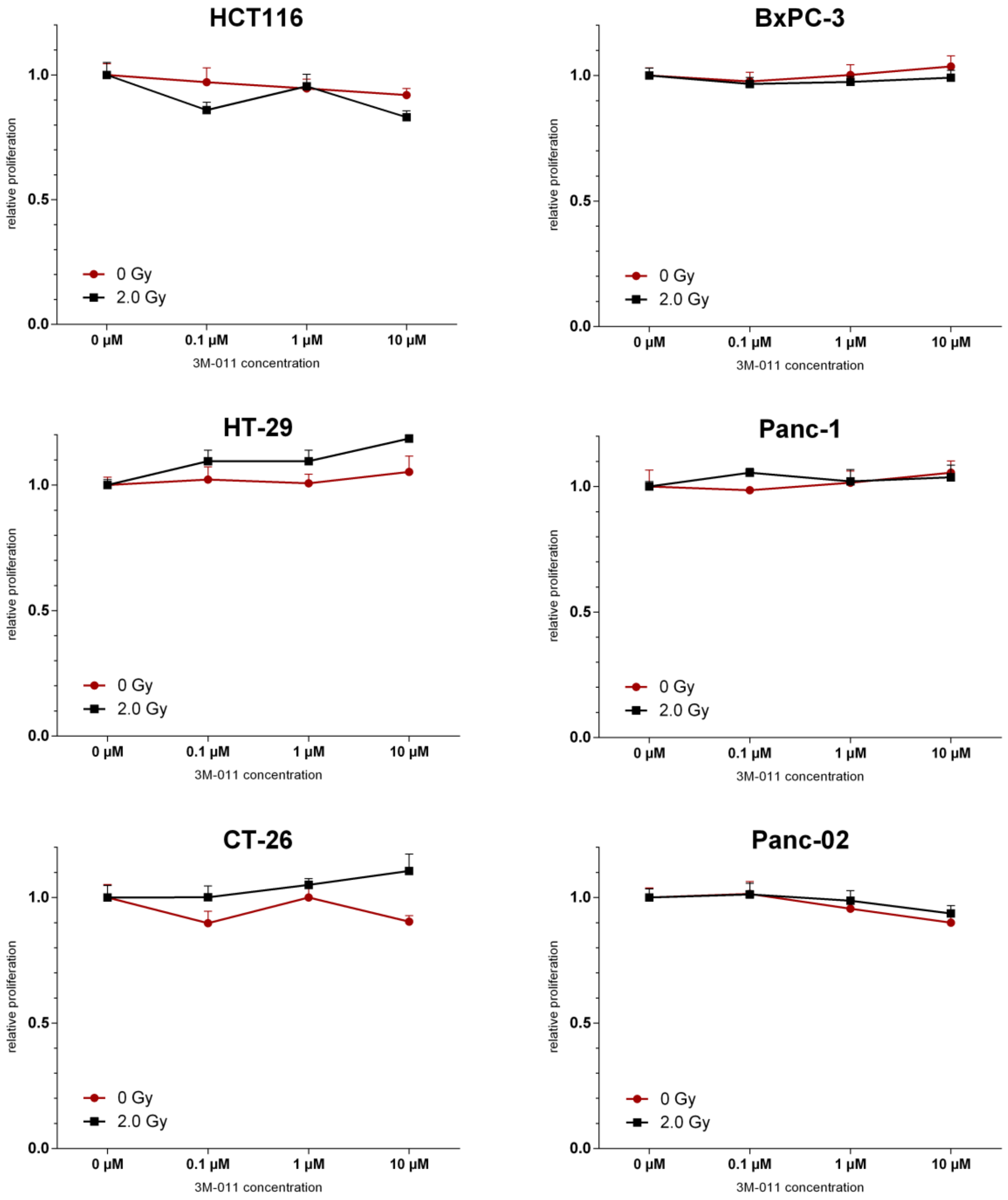

Supplemental Fig. 1: WST proliferation assays of CRC (left graphs) and PDAC (right graphs) cell lines incubated with increasing concentrations of 3M-011. The 2 top rows are human cell lines, the bottom row are murine cell lines. *All values represent mean ± SEM.*

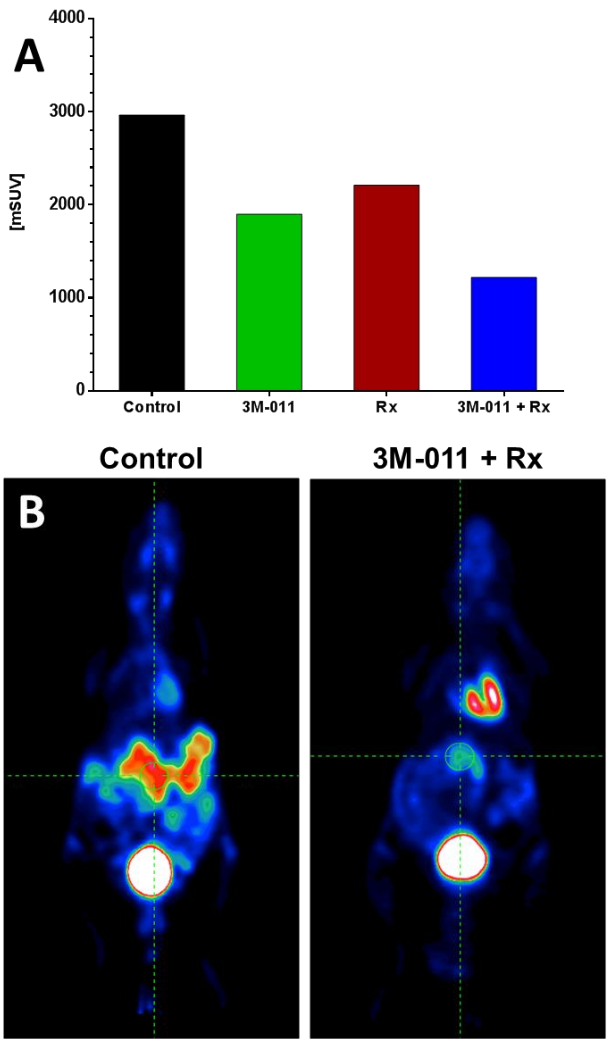

Supplemental Fig. 2: A. Tumor standardized uptake value (SUV) of FDG-PET imaging of mice bearing orthotopic Panc-02 homografts at day 11 after tumor inoculation). **B:** Exemplary coronal FDG-PET images of the same mice (crosslines indicate the tumor-related FDG uptake).

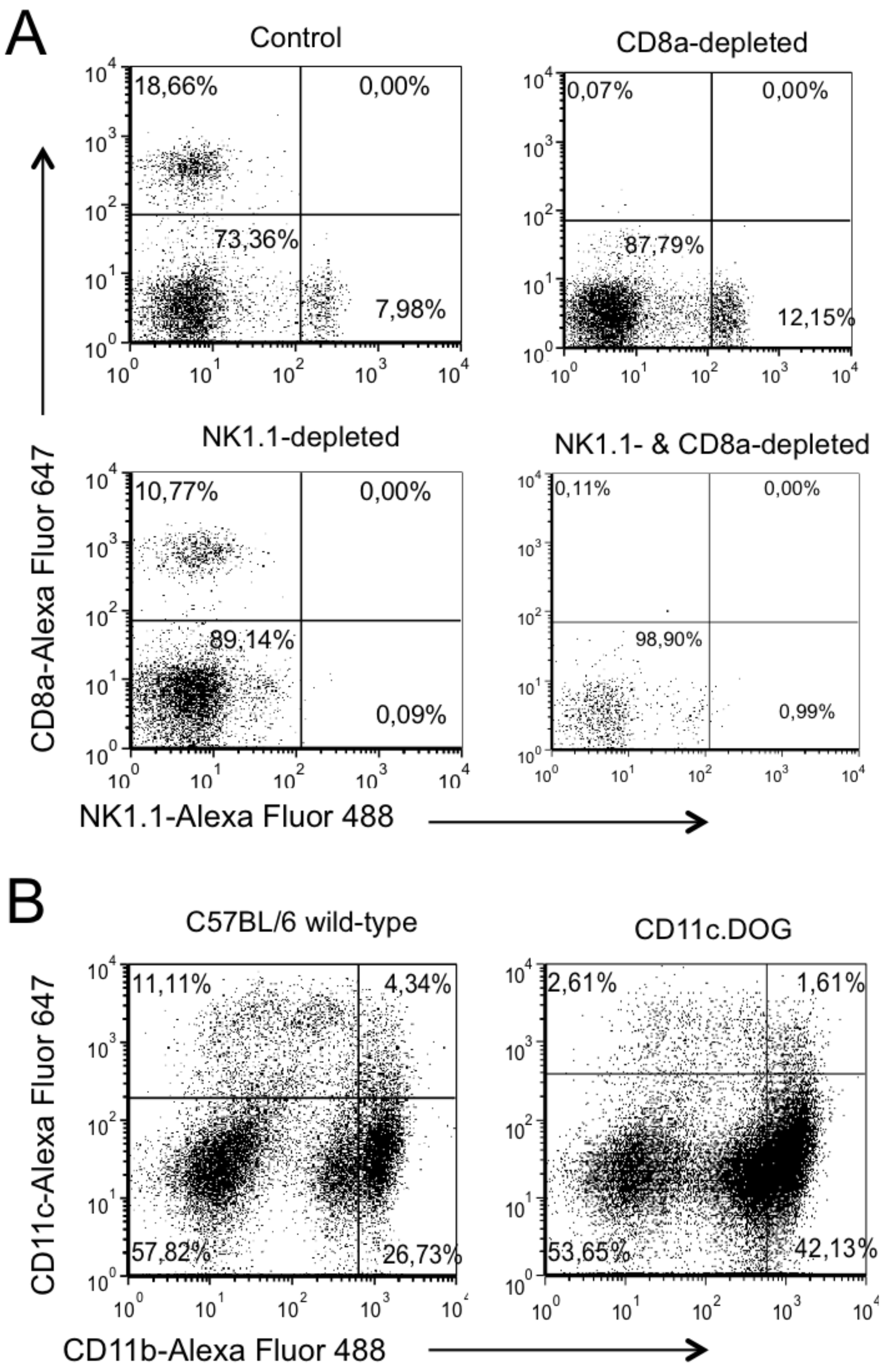

Supplemental Fig. 3: A: Exemplary FACS plots depicting successful antibody-mediated NK and CD8 T cell depletion in mouse PBMC. **B.** Exemplary FACS plots of splenocytes depicting successful depletion of CD11c+ dendritic cells by DT injection in CD11c-DOG mice.

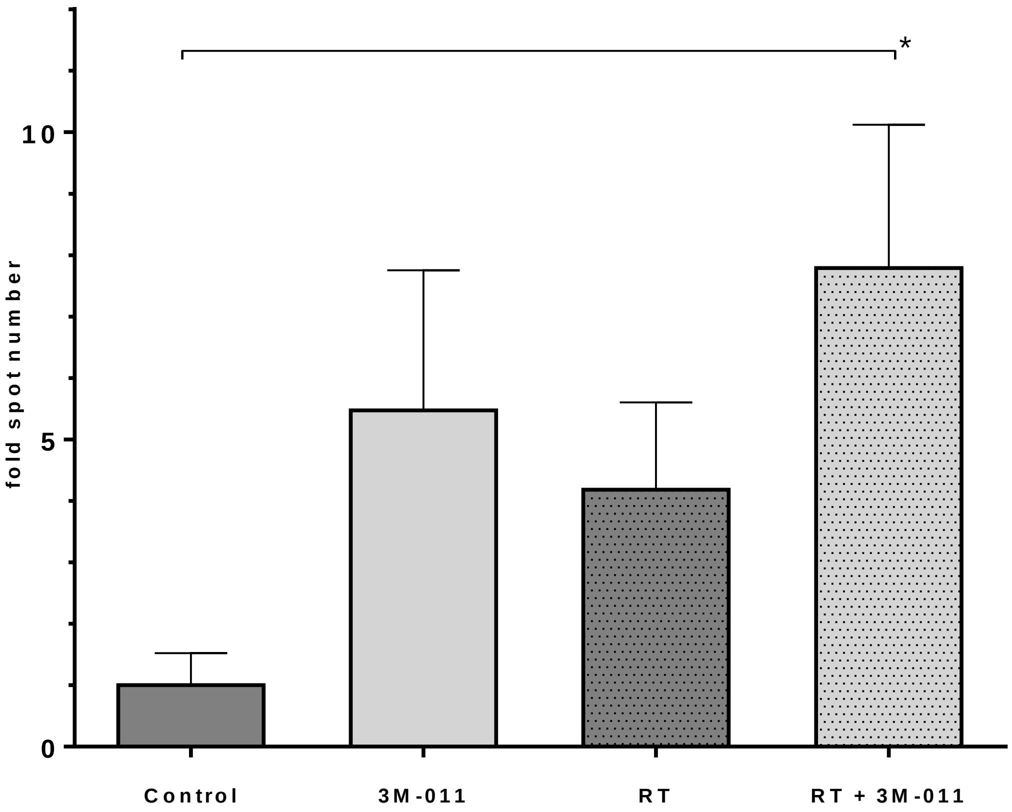

Supplemental Fig. 4: ELISPOT assay investigating CD90[+] splenocyte reactivity to AH-1 peptide in mice bearing CT26 homografts and treated as indicated. The y axis represents the number of spots, normalized to the control. *All values represent mean ± SEM.*

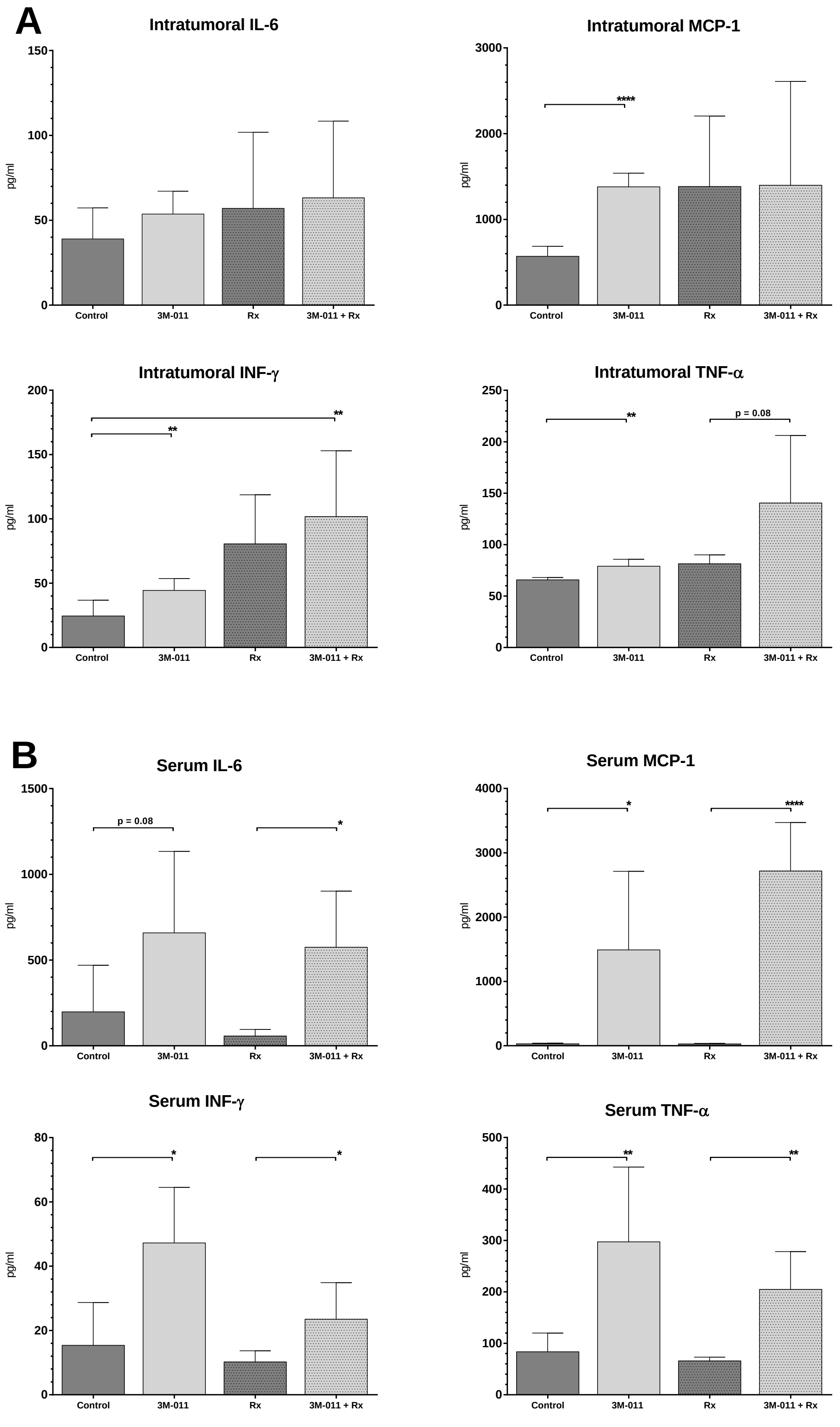

Supplemental Fig. 5: Intratumoral (A) and serum (B) cytokine levels. *All values represent mean ± SEM.*
